# Supplementary material for: Profiling Plasma Cytokines by A CRISPR-ELISA Assay for Early Detection of Lung Cancer
Source: J Clin Med. 2022 Nov 24;11(23):6923. doi: 10.3390/jcm11236923 (PMC9740838; doi:10.3390/jcm11236923)
Supplement: Supplementary file 1 [file jcm-11-06923-s001.zip › jcm-2036984-supplementary.pdf]

Supplementary Table S1. Day to day reproducibility of the CRSPR-ELISA assay for detection of cytokines

Cytokines	*CV (%)
IL-1b	9.257
IL-2	7.632
IL-6	11.731
IL-7	10.489
IL-8	11.307
IL-10	5.657
IL-12p70	6.535
IL-13	8.535
IL-17A	5.654
MCP1	9.554
IFN-γ	12.455
TNF-α	13.646

*, CV, coefficient of variation.

Supplementary Table S2. Inter-assay precision of the CRSPR-ELISA assay detection of cytokines

Cytokines	CV (%)
IL-1b	11.436
IL-2	7.652
IL-6	12.786
IL-7	10.685
IL-8	9.646
IL-10	11.864
IL-12p70	7.656
IL-13	6.764
IL-17A	12.757
MCP1	9.575
IFN-γ	12.675
TNF-α	8.646

*, CV, coefficient of variation.

Supplementary Table S3. Intra-assay precision of the CRSPR-ELISA assay detection of cytokines

Cytokines	CV (%)
IL-1b	8.127
IL-2	10.468
IL-6	7.965
IL-7	11.654
IL-8	7.544
IL-10	9.945
IL-12p70	10.435
IL-13	6.674
IL-17A	13.854
MCP1	11.468
IFN-γ	9.945

TNF-α	11.673

*, CV, coefficient of variation.
